# Supplementary material for: A cell type-aware framework for nominating non-coding variants in Mendelian regulatory disorders
Source: Nat Commun. 2024 Sep 27;15:8268. doi: 10.1038/s41467-024-52463-7 (PMC11436875; doi:10.1038/s41467-024-52463-7)
Supplement: Supplementary file 1 — Supplementary Information [file 41467_2024_52463_MOESM1_ESM.pdf]

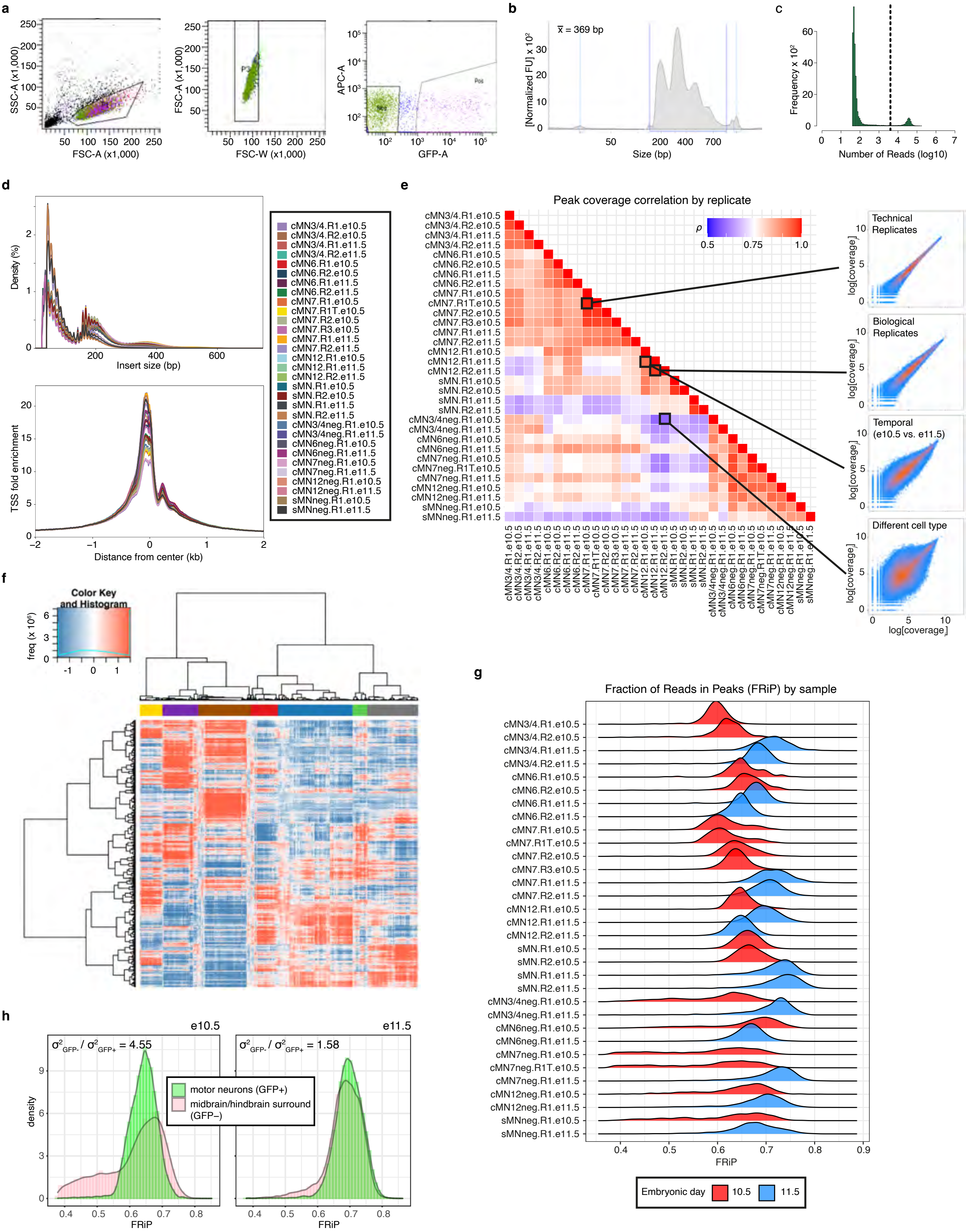
a
SSC-A (x1,000)
FSC-A (x1,000)
FSC-W (x1,000)
P3
APC-A
GFP-A
Pos
b
x̄ = 369 bp
[Normalized FU] x 10²
Size (bp)
c
Frequency x 10²
Number of Reads (log10)
d
Density (%)
Insert size (bp)
TSS fold enrichment
Distance from center (kb)
cMN3/4.R1.e10.5
cMN3/4.R2.e10.5
cMN3/4.R1.e11.5
cMN3/4.R2.e11.5
cMN6.R1.e10.5
cMN6.R2.e10.5
cMN6.R1.e11.5
cMN6.R2.e11.5
cMN7.R1.e10.5
cMN7.R1T.e10.5
cMN7.R2.e10.5
cMN7.R3.e10.5
cMN7.R1.e11.5
cMN7.R2.e11.5
cMN12.R1.e10.5
cMN12.R1.e11.5
cMN12.R2.e11.5
sMN.R1.e10.5
sMN.R2.e10.5
sMN.R1.e11.5
sMN.R2.e11.5
cMN3/4neg.R1.e10.5
cMN3/4neg.R1.e11.5
cMN6neg.R1.e10.5
cMN6neg.R1.e11.5
cMN7neg.R1.e10.5
cMN7neg.R1.e11.5
cMN12neg.R1.e10.5
cMN12neg.R1.e11.5
sMNneg.R1.e10.5
sMNneg.R1.e11.5
e
Peak coverage correlation by replicate
ρ
Technical Replicates
Biological Replicates
Temporal (e10.5 vs. e11.5)
Different cell type
log[coverage]
f
Color Key and Histogram
freq (x 10⁶)
g
Fraction of Reads in Peaks (FRiP) by sample
FRiP
Embryonic day
10.5
11.5
h
e10.5
e11.5
σ²GFP- / σ²GFP+ = 4.55
σ²GFP- / σ²GFP+ = 1.58
density
motor neurons (GFP+)
midbrain/hindbrain surround (GFP−)
FRiP

Supplementary Figure 1. Per-cell and -sample quality metrics for scATAC data.

a. Representative FACS gating strategy for WT GFP-positive and GFP-negative cMN7 at e10.5. Left: Forward scatter area (FSC-A) and side scatter area (SSC-A), corresponding to cell size and granularity/complexity, are used to enrich for intact cells and exclude debris. Middle: forward scatter width (FSC-W) and FSC-A are used to exclude doublets. Right: Green fluorescent protein area (GFP-A) and 633 nm-excitation (APC-A) are used to enrich for GFP-positive and GFP-negative cells. GFP-negative gates are calibrated by dissociated limb buds prior to collection as a negative control. All samples are fresh, live cells without fixative or nuclear staining.
b. Representative TapeStation trace showing tagmented DNA fragment sizes prior to library preparation.
c. Representative histogram of per-cell scATAC reads in a single sample. Read cutoff is shown by a dotted line and determined heuristically for each sample.
d. Insert size distributions (top) and transcriptional start site (TSS) enrichment (bottom) for all samples and replicates. Insert sizes consistently show a characteristic nucleosome banding pattern (~147 bp wavelength). Samples IDs are shown in **Supplementary Data**.
e. Correlation matrix depicting all possible pairwise sample correlations (Spearman's rho) for scATAC coverage in all rank-ordered peaks. Scatterplots for selected sample pairs from the four highlighted boxes within the matrix are shown on the right. Correlations decrease with increasing biological distance (top to bottom).
f. Representative clade diagram depicting the relative accessibility (red is positive, blue is negative) of 5kb genomic windows (rows) across individual cells within a given sample (columns). Distinct clades (colored bars) were determined heuristically for each sample for downstream peak calling. The number of clades per sample were selected to maximize representation of common and rare cell types.
g. Ridgeplot depicting density of per-cell fraction of reads in peaks (FRiP) for each dissected sample and replicate at e10.5 (red) and e11.5 (blue). Samples IDs are shown in **Supplementary Data**. Mean FRiP values are consistently higher for e11.5 samples (p-value = 4×10^{-5}, binomial test).
h. Distribution of FRiP values for GFP-positive motor neurons (green) versus GFP-negative surrounding brain tissue (pink). GFP-negative cells display significantly greater dispersion compared to GFP-positive cells, particularly at e10.5. (p-value = 1.1×10^{-286}, Brown-Forsythe Test). See **Supplementary Note 1** for additional information.

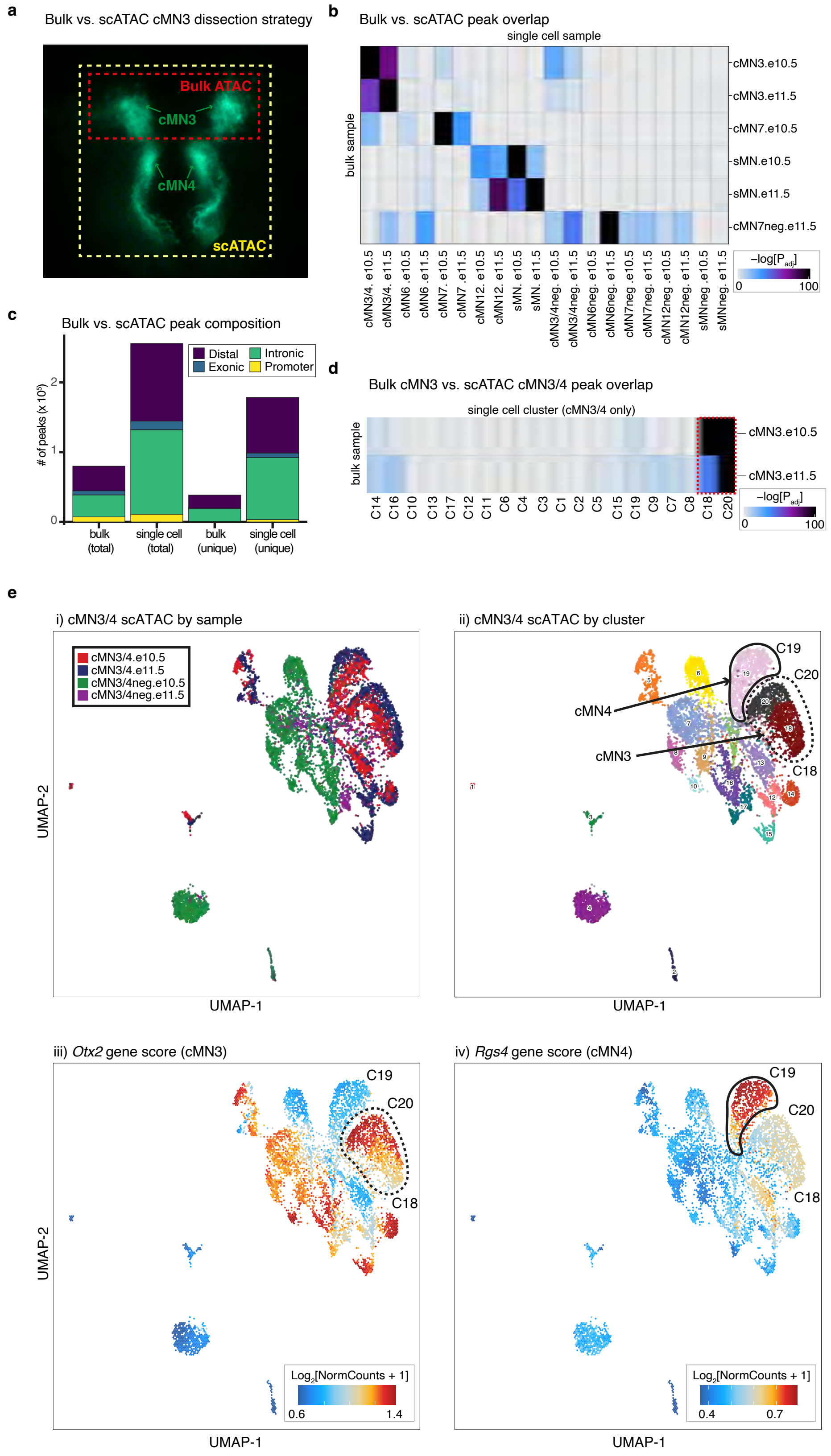

a
Bulk vs. scATAC cMN3 dissection strategy
Bulk ATAC
cMN3
cMN4
scATAC
b
Bulk vs. scATAC peak overlap
single cell sample
bulk sample
cMN3.e10.5
cMN3.e11.5
cMN7.e10.5
sMN.e10.5
sMN.e11.5
cMN7neg.e11.5
cMN3/4. e10.5
cMN3/4. e11.5
cMN6. e10.5
cMN6. e11.5
cMN7. e10.5
cMN7 .e11.5
cMN12. e10.5
cMN12. e11.5
sMN. e10.5
sMN. e11.5
cMN3/4neg. e10.5
cMN3/4neg. e11.5
cMN6neg. e10.5
cMN6neg. e11.5
cMN7neg .e10.5
cMN7neg. e11.5
cMN12neg .e10.5
cMN12neg. e11.5
sMNneg. e10.5
sMNneg. e11.5
−log[P adj]
0
100
c
Bulk vs. scATAC peak composition
Distal
Intronic
Exonic
Promoter
# of peaks (x 10⁵)
0
1
2
bulk (total)
single cell (total)
bulk (unique)
single cell (unique)
d
Bulk cMN3 vs. scATAC cMN3/4 peak overlap
single cell cluster (cMN3/4 only)
bulk sample
cMN3.e10.5
cMN3.e11.5
C14
C16
C10
C13
C17
C12
C11
C6
C4
C3
C1
C2
C5
C15
C19
C9
C7
C8
C18
C20
−log[P adj]
0
100
e
i) cMN3/4 scATAC by sample
cMN3/4.e10.5
cMN3/4.e11.5
cMN3/4neg.e10.5
cMN3/4neg.e11.5
UMAP-2
UMAP-1
ii) cMN3/4 scATAC by cluster
C19
C20
cMN4
cMN3
C18
UMAP-1
iii) Otx2 gene score (cMN3)
C19
C20
C18
UMAP-2
Log2[NormCounts + 1]
0.6
1.4
UMAP-1
iv) Rgs4 gene score (cMN4)
C19
C20
C18
Log2[NormCounts + 1]
0.4
0.7
UMAP-1

Supplementary Figure 2. Comparing and contrasting bulk versus single cell ATAC profiles.

a. Fluorescence microscopy image illustrating cMN3 and cMN4 microdissection strategies. For scATAC experiments, cMN3 and cMN4 were microdissected *en bloc* (yellow box). For bulk ATAC microdissections, only cMN3 was excised (red box). All other cMN microdissection strategies were identical across bulk and scATAC.
b. Heatmap depicting enrichment of sample-specific bulk ATAC versus scATAC peaks. Color scale represents hypergeometric test p-values using the *peakAnnoEnrichment()* function in *ArchR*. Samples marked with "neg" are GFP-negative cells surrounding the motor neurons of interest. All other samples are GFP-positive motor neurons.
c. Stacked barplot depicting relative proportions of different classes of accessible chromatin ("distal", "exonic", "intronic", and "promoter"). scATAC peaks are enriched for total number of peaks, total number of unique peaks, and cell type-specific peak annotations (distal and intronic).
d. Heatmap depicting enrichment of overlapping peaks for bulk cMN3 dissections versus *ad hoc* clusters (C1-C20) generated from scATAC cMN3/4 dissections only. Color scale represents hypergeometric test p-values*. Ad hoc* clusters C18 and C20 with the highest peak enrichment for bulk cMN3 are outlined by dashed red lines.
e. *In silico* microdissection of scATAC cMN3/4 clusters corroborates physical microdissections. Left to right, UMAP embeddings of scATAC cMN3/4 dissections colored by i) dissected sample; ii) *ad hoc* clusters; and gene scores for iii) cMN3 marker gene *Otx2* (https://doi.org/10.1016/0925-4773(95)00421-1); and iv) cMN4 marker gene *Rgs4* (https://doi.org/10.1523/jneurosci.23-33-10613.2003). Putative cMN3 (C18 and C20) and cMN4 (C19) clusters inferred from dissection origin, marker genes, and GFP status are denoted by dashed and solid red lines, respectively.

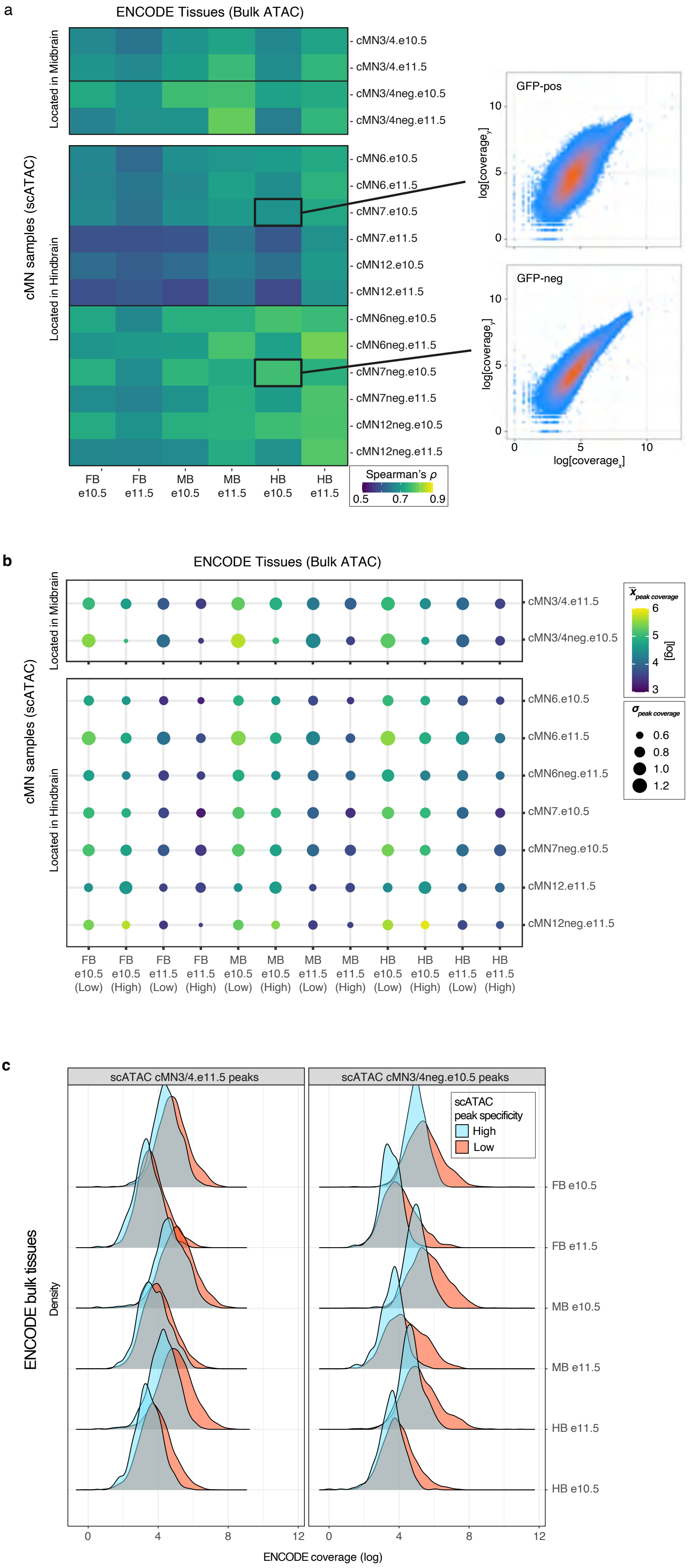

a
ENCODE Tissues (Bulk ATAC)
cMN samples (scATAC)
Located in Midbrain
Located in Hindbrain
cMN3/4.e10.5
cMN3/4.e11.5
cMN3/4neg.e10.5
cMN3/4neg.e11.5
cMN6.e10.5
cMN6.e11.5
cMN7.e10.5
cMN7.e11.5
cMN12.e10.5
cMN12.e11.5
cMN6neg.e10.5
cMN6neg.e11.5
cMN7neg.e10.5
cMN7neg.e11.5
cMN12neg.e10.5
cMN12neg.e11.5
FB e10.5
FB e11.5
MB e10.5
MB e11.5
HB e10.5
HB e11.5
Spearman's ρ
0.5 0.7 0.9
GFP-pos
GFP-neg
log[coverage_y]
log[coverage_x]
b
ENCODE Tissues (Bulk ATAC)
cMN samples (scATAC)
Located in Midbrain
Located in Hindbrain
cMN3/4.e11.5
cMN3/4neg.e10.5
cMN6.e10.5
cMN6.e11.5
cMN6neg.e11.5
cMN7.e10.5
cMN7neg.e10.5
cMN12.e11.5
cMN12neg.e11.5
FB e10.5 (Low)
FB e10.5 (High)
FB e11.5 (Low)
FB e11.5 (High)
MB e10.5 (Low)
MB e10.5 (High)
MB e11.5 (Low)
MB e11.5 (High)
HB e10.5 (Low)
HB e10.5 (High)
HB e11.5 (Low)
HB e11.5 (High)
x̄ peak coverage
[log]
σ peak coverage
0.6
0.8
1.0
1.2
c
scATAC cMN3/4.e11.5 peaks
scATAC cMN3/4neg.e10.5 peaks
scATAC peak specificity
High
Low
FB e10.5
FB e11.5
MB e10.5
MB e11.5
HB e11.5
HB e10.5
ENCODE bulk tissues
Density
ENCODE coverage (log)

Supplementary Figure 3. Cranial motor neuron scATAC peaks are underrepresented in regional bulk datasets.

a. (Left) Heatmap depicting correlation coefficients (Spearman's *ρ*) between scATAC peaks from cMN microdissections versus bulk ATAC peaks from ENCODE e10.5 and e11.5 mouse developing forebrain (FB), midbrain (MB), and hindbrain (HB) dissections. Anatomically concordant bulk brain regions are more highly correlated with scATAC non-motor neuron samples ('-neg') than scATAC cranial motor neuron samples. (Right) Scatterplots depicting rank-ordered per-peak sequencing coverage for bulk vs. scATAC samples.
b. Bubble chart depicting ENCODE bulk ATAC coverage in scATAC cMN peaks from a subset of samples, stratified by cell type specificity scores ('High' vs. 'Low'). Colors reflect mean peak coverage (with lighter color reflecting higher coverage), while area reflects standard deviation. Bulk tissues tend to have higher coverage in low specificity peaks when compared to highly cell type specific peaks.
c. Density plots depicting distribution of ENCODE bulk peak coverage within cMN3/4 scATAC peaks from (b), stratified by specificity scores. High specificity scATAC peaks (blue) have consistently lower bulk coverage compared to low specificity peaks (red).

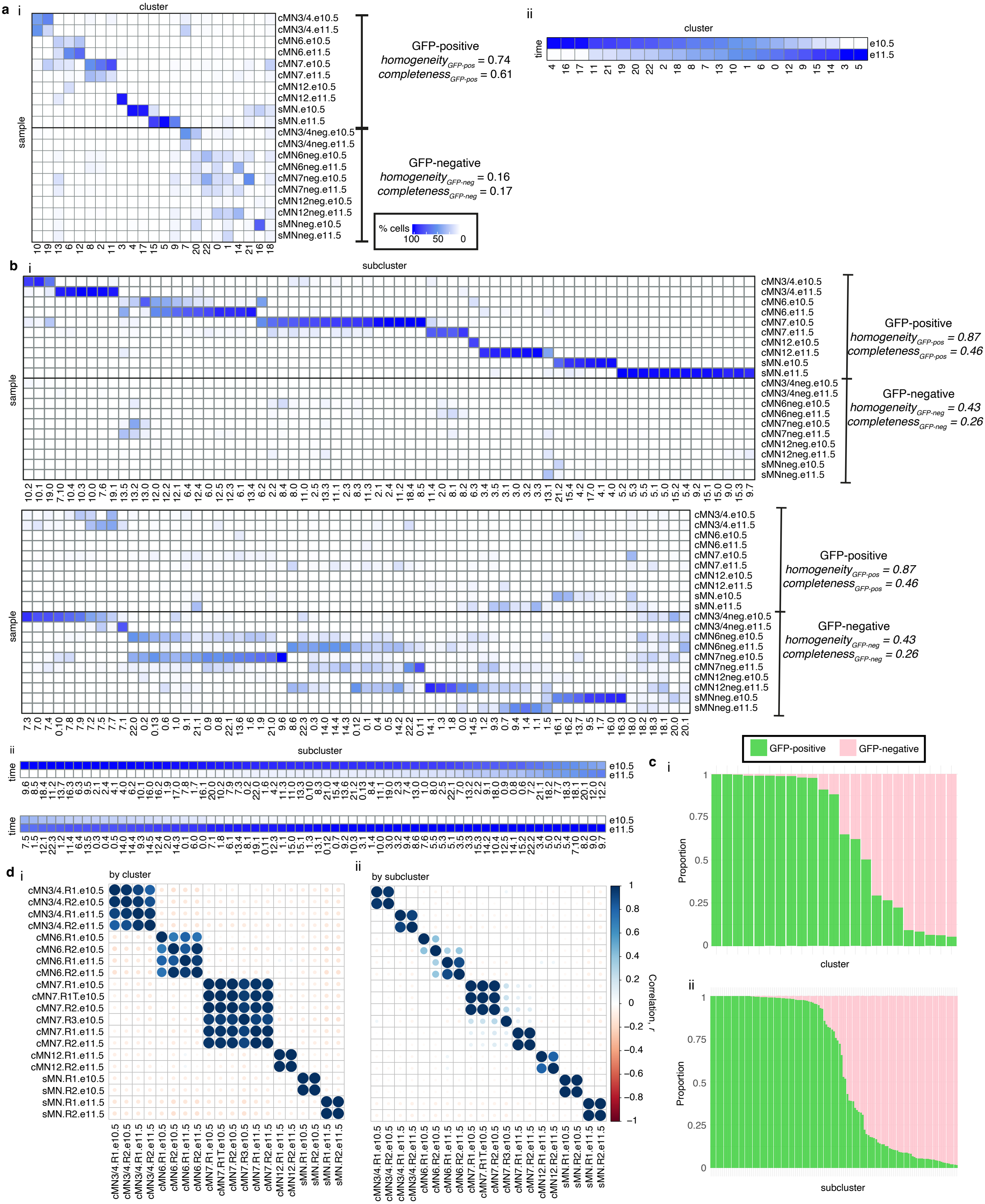

a
i
cluster
sample
cMN3/4.e10.5
cMN3/4.e11.5
cMN6.e10.5
cMN6.e11.5
cMN7.e10.5
cMN7.e11.5
cMN12.e10.5
cMN12.e11.5
sMN.e10.5
sMN.e11.5
cMN3/4neg.e10.5
cMN3/4neg.e11.5
cMN6neg.e10.5
cMN6neg.e11.5
cMN7neg.e10.5
cMN7neg.e11.5
cMN12neg.e10.5
cMN12neg.e11.5
sMNneg.e10.5
sMNneg.e11.5
10 19 13 6 12 8 2 11 3 4 17 15 5 9 7 20 22 0 1 14 21 16 18
GFP-positive
homogeneity GFP-pos = 0.74
completeness GFP-pos = 0.61
GFP-negative
homogeneity GFP-neg = 0.16
completeness GFP-neg = 0.17
% cells
100 50 0
ii
cluster
time
e10.5
e11.5
4 16 17 11 21 19 20 22 2 18 8 7 13 10 1 6 0 12 9 15 14 3 5
b
i
subcluster
sample
GFP-positive
homogeneity GFP-pos = 0.87
completeness GFP-pos = 0.46
GFP-negative
homogeneity GFP-neg = 0.43
completeness GFP-neg = 0.26
ii
subcluster
time
e10.5
e11.5
c
i
GFP-positive
GFP-negative
Proportion
1 0.75 0.5 0.25 0
cluster
ii
Proportion
subcluster
d
i
by cluster
cMN3/4.R1.e10.5
cMN3/4.R2.e10.5
cMN3/4.R1.e11.5
cMN3/4.R2.e11.5
cMN6.R1.e10.5
cMN6.R2.e10.5
cMN6.R1.e11.5
cMN6.R2.e11.5
cMN7.R1.e10.5
cMN7.R1T.e10.5
cMN7.R2.e10.5
cMN7.R3.e10.5
cMN7.R1.e11.5
cMN7.R2.e11.5
cMN12.R1.e11.5
cMN12.R2.e11.5
sMN.R1.e10.5
sMN.R2.e10.5
sMN.R1.e11.5
sMN.R2.e11.5
ii
by subcluster
Correlation, r
1 0.8 0.6 0.4 0.2 0 −0.2 −0.4 −0.6 −0.8 −1

Supplementary Figure 4. scATAC cluster purity across major clusters and subclusters.

a. Heatmaps depicting purity of the 23 major scATAC clusters, stratified by i) sample and ii) embryonic age. cMN7 cells migrate past cMN6, are in close spatial proximity at these developmental ages, and are commonly co-dissected. Samples are GFP-positive unless otherwise marked ('neg'). Clusters with higher membership from GFP-positive samples have higher purity than clusters with higher membership from GFP-negative samples. Most clusters feature cells from both e10.5 and e11.5 dissections, consistent with ongoing cell birth and proliferation. Homogeneity/completeness metrics calculated for GFP-positive versus GFP-negative samples are shown.
b. Heatmaps depicting purity of the 132 scATAC subclusters, stratified by i) sample and ii) embryonic age. As observed with the major clusters in (a), subclusters with high GFP-positive membership have greater purity than high GFP-negative subclusters. In contrast to the major clusters, a greater proportion of subclusters have skewed temporal membership (e10.5 vs. e11.5), potentially reflecting transient cell states.
c. Stacked barplots depicting proportion of GFP-positive and -negative cells within each i) cluster and ii) subcluster. Most clusters and subclusters are skewed towards pure (i.e., > 90%) GFP-positive or -negative membership. Here Cluster/subcluster IDs are not shown for ease of visualization. Detailed cluster annotations are available in **Supplementary Data**.
d. Correlation matrix depicting pairwise correlations between all biological replicates among i) major clusters and ii) subclusters. Cluster/subcluster membership is highly correlated across biological replicates from different batches, particularly for subclusters.

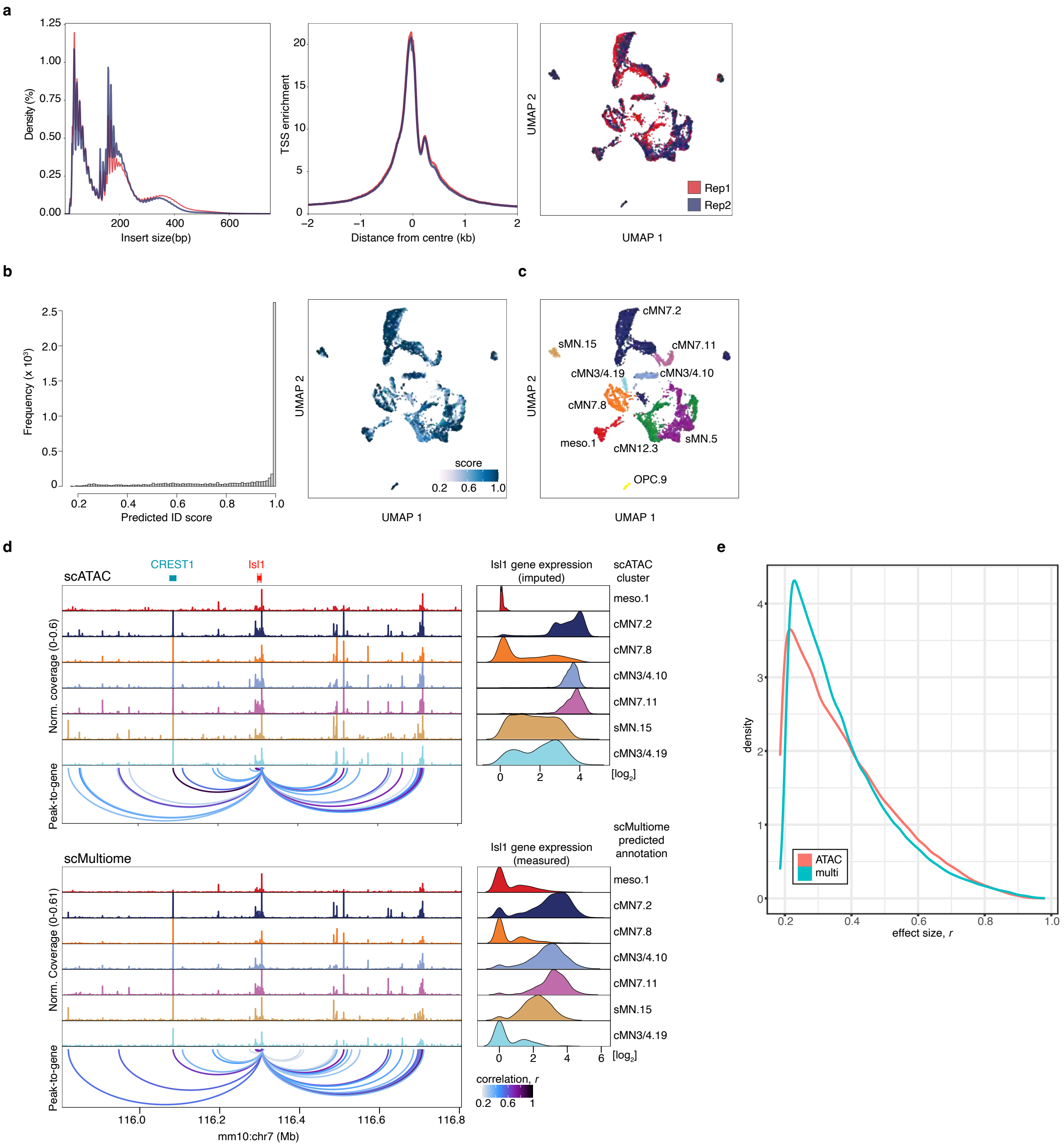
a
Density (%)
Insert size(bp)
TSS enrichment
Distance from centre (kb)
UMAP 2
UMAP 1
Rep1
Rep2
b
Frequency (x 10³)
Predicted ID score
score
c
cMN7.2
sMN.15
cMN7.11
cMN3/4.19
cMN3/4.10
cMN7.8
meso.1
cMN12.3
sMN.5
OPC.9
d
scATAC
CREST1
Isl1
Isl1 gene expression (imputed)
scATAC cluster
Norm. coverage (0-0.6)
Peak-to-gene
[log2]
scMultiome
Isl1 gene expression (measured)
scMultiome predicted annotation
Norm. Coverage (0-0.61)
correlation, r
mm10:chr7 (Mb)
e
density
effect size, r
ATAC
multi

Supplementary Figure 5. Single cell multiome reproducibility and quality control.

a. Chromatin fragment length distribution (left), transcription start site (TSS) enrichment (middle), and joint UMAP embedding (right) comparing scMultiome biological replicates (red and blue). Replicates are highly concordant.
b. Histogram (left) and UMAP embedding (right) depicting distribution of scMultiome prediction ID scores of annotations transferred from the scATAC reference set to the scMultiome query set using the *TransferData()* function in Seurat(https://doi.org/10.1016/j.cell.2019.05.031). The distribution is heavily skewed towards higher scores.
c. scMultiome annotations based on prediction IDs. Most predicted annotations correspond to *Isl1*^{MN}:GFP-positive cell types, consistent with scMultiome dissection strategy.
d. Direct comparison of peak-to-gene links from scATAC versus scMultiome for motor neuron master regulator *Isl1*. scATAC peak-to-gene links are generated from imputed gene expression values ("GeneIntegrationMatrix") whereas scMultiome links are generated from direct gene expression measurements ("GeneExpressionMatrix"). Ground truth enhancer CREST1 is highly accessible in *Isl1*-positive clusters with strong peak-to-gene links across both modalities.
e. Distribution of peak-to-gene effect sizes from scATAC versus scMultiome across the genome (shared links only).

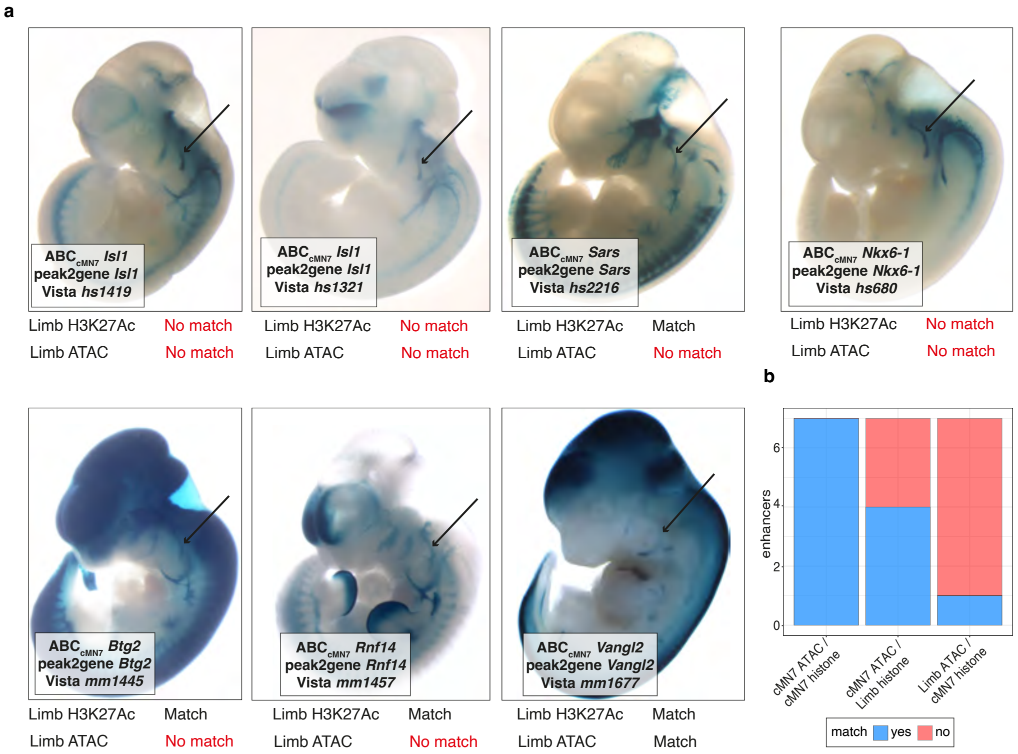

a
ABC cMN7 Isl1
peak2gene Isl1
Vista hs1419
Limb H3K27Ac No match
Limb ATAC No match
ABC cMN7 Isl1
peak2gene Isl1
Vista hs1321
Limb H3K27Ac No match
Limb ATAC No match
ABC cMN7 Sars
peak2gene Sars
Vista hs2216
Limb H3K27Ac Match
Limb ATAC No match
ABC cMN7 Nkx6-1
peak2gene Nkx6-1
Vista hs680
Limb H3K27Ac No match
Limb ATAC No match
ABC cMN7 Btg2
peak2gene Btg2
Vista mm1445
Limb H3K27Ac Match
Limb ATAC No match
ABC cMN7 Rnf14
peak2gene Rnf14
Vista mm1457
Limb H3K27Ac Match
Limb ATAC No match
ABC cMN7 Vangl2
peak2gene Vangl2
Vista mm1677
Limb H3K27Ac Match
Limb ATAC Match
b
enhancers
0
2
4
6
cMN7 ATAC / cMN7 histone
cMN7 ATAC / Limb histone
Limb ATAC / cMN7 histone
match yes no

Supplementary Figure 6. Toggling input data for Activity-by-Contact enhancer prediction.

a. Whole mount *in vivo* enhancer reporter expression for the seven VISTA Enhancers that are annotated for cranial nerve (CN) expression, inspected for and have CN7 expression (https://doi.org/10.1016/j.gep.2013.07.001), and have positive Activity-by-Contact (ABC) enhancer predictions for CN7 at e11.5. Peak-to-gene predictions match ABC predictions in all cases (7/7 enhancers). Replacing CN7 e11.5 H3K27Ac or ATAC data with these data from a distantly related cell type (mouse embryonic limb e11.5) results in either a matching or a non-matching cognate gene prediction. Substituting cMN7 e11.5 histone modification data with "Limb H3K27Ac" histone modification data alters predictions for 3 out of 7 enhancers. Substituting cMN7 scATAC data with "Limb ATAC" data alters predictions for 6 out of 7 enhancers. Neither substituted input correctly identifies the CREST1 enhancer (VISTA enhancer hs1419). Positive evidence of CN7 enhancement is depicted by arrows.
b. Stacked barplot summarizing consequences of toggled input data.

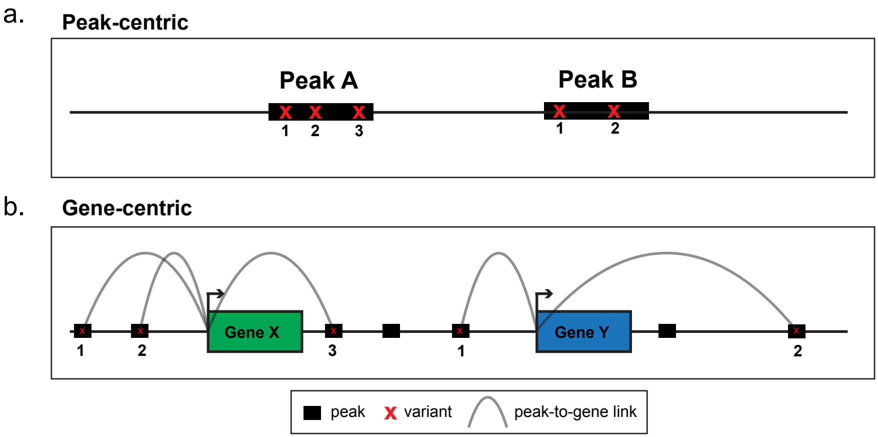

a.
Peak-centric
Peak A
1
2
3
Peak B
1
2
b.
Gene-centric
Gene X
Gene Y
1
2
3
1
2
peak
variant
peak-to-gene link

Supplementary Figure 7. Peak- and gene-centric aggregation approaches.

Illustration depicting a) peak- and b) gene-centric aggregation approaches. The peak-centric approach aggregates variants that overlapping shared peaks. Conversely, the gene-centric approach aggregates variants overlapping peaks with a shared target gene specified by peak-to-gene links.

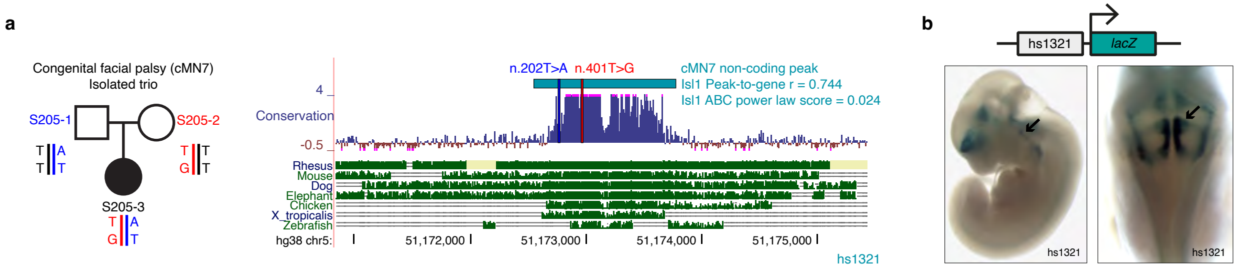
a
Congenital facial palsy (cMN7)
Isolated trio
S205-1
S205-2
S205-3
n.202T>A
n.401T>G
cMN7 non-coding peak
Isl1 Peak-to-gene r = 0.744
Isl1 ABC power law score = 0.024
Conservation
4
-0.5
Rhesus
Mouse
Dog
Elephant
Chicken
X_tropicalis
Zebrafish
hg38 chr5:
51,172,000
51,173,000
51,174,000
51,175,000
hs1321
b
hs1321
lacZ
hs1321
hs1321

Supplementary Figure 8. Compound heterozygous non-coding candidate variants in an *ISL1* enhancer.

a. An affected trio with isolated congenital facial palsy, a CCDD affecting cMN7 (left), in which the affected offspring harbors compound heterozygous non-coding candidate SNVs (depicted by blue and red bars) affecting highly conserved nucleotides in enhancer hs1321 (right). The enhancer is predicted to regulate *Isl1* (peak-to-gene r = 0.744, ABC power law = 0.024). Variant coordinates are in NG_023040.1.
b. *In vivo* reporter assay testing hs1321 enhancer activity (n=5). Enhancement is present in cranial nerve 7 (arrows), an *Isl1* positive cell type. Reporter expression views are shown as lateral (left) and dorsal through the 4th ventricle (right).

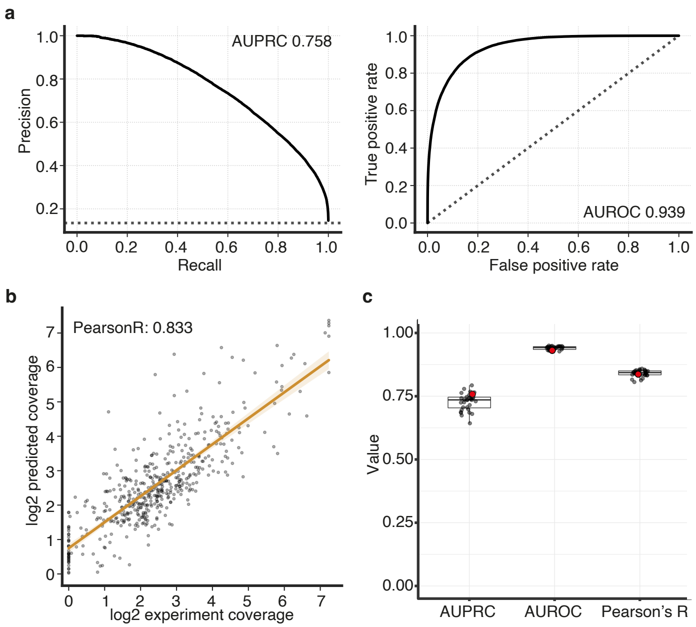
a
AUPRC 0.758
Precision
Recall
AUROC 0.939
True positive rate
False positive rate
b
PearsonR: 0.833
log2 predicted coverage
log2 experiment coverage
c
Value
AUPRC
AUROC
Pearson's R

Supplementary Figure 9. Quality metrics for *Basenji* convolutional neural network accessibility predictions.

a. Precision-recall (PRC, left) and receiver-operating characteristic (ROC, right) curves measuring favorable performance (as measured by positive predictive value, sensitivity, true positive rate, and false positive rate) of *Basenji* accessibility predictions for cMN7 e10.5. AU denotes area under curve. Dotted lines represent the baseline classification rate.
b. Scatterplot depicting *Basenji* accessibility predictions vs. true scATAC sequencing coverage for cMN7 e10.5. Each point represents a 128 bp test bin whose sequence was excluded from training. Measured and predicted coverage are positively correlated (Pearson's R = 0.833).
c. Boxplot summarizing area under PRC (AUPRC) and ROC (AUROC), and Pearson's R for all samples and replicates. Quality metrics are consistent across samples. Data points depicted in (a) and (b) are highlighted in red. Centre line – median; box limits – upper and lower quartiles; whiskers – 1.5 x interquartile range.

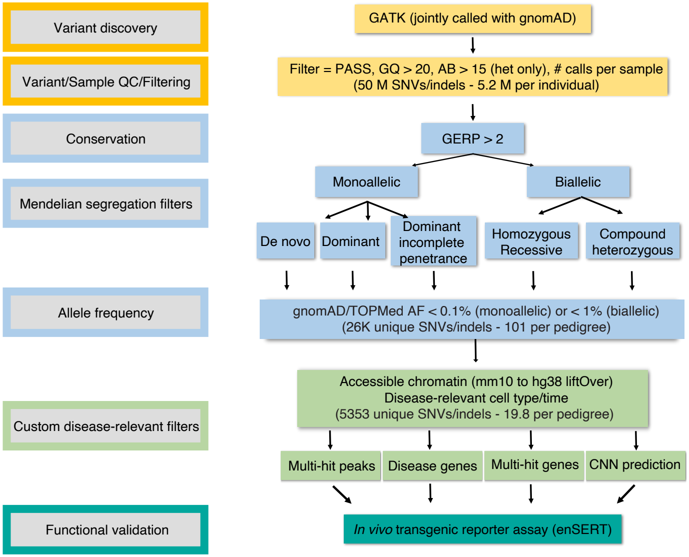

Variant discovery
Variant/Sample QC/Filtering
Conservation
Mendelian segregation filters
Allele frequency
Custom disease-relevant filters
Functional validation
GATK (jointly called with gnomAD)
Filter = PASS, GQ > 20, AB > 15 (het only), # calls per sample
(50 M SNVs/indels - 5.2 M per individual)
GERP > 2
Monoallelic
Biallelic
De novo
Dominant
Dominant incomplete penetrance
Homozygous Recessive
Compound heterozygous
gnomAD/TOPMed AF < 0.1% (monoallelic) or < 1% (biallelic)
(26K unique SNVs/indels - 101 per pedigree)
Accessible chromatin (mm10 to hg38 liftOver)
Disease-relevant cell type/time
(5353 unique SNVs/indels - 19.8 per pedigree)
Multi-hit peaks
Disease genes
Multi-hit genes
CNN prediction
In vivo transgenic reporter assay (enSERT)

Supplementary Figure 10. Variant filtering and prioritization workflow for functional validation.

Flowchart depicting variant filtering steps for selecting targets for functional validation. Jointly genotyped variants are QC'd, filtered for conservation, Mendelian segregation, and allele frequency. Custom disease filters are applied based on chromatin accessibility in orthologous mouse sequence. Multiple prioritization strategies (multi-hit peaks, peak-to-gene connections to disease genes, multiple peak-to-gene connections to any genes, and machine learning predictions) are used to select individual enhancers and variants for *in vivo* functional validation. CNN – convolutional neural network.

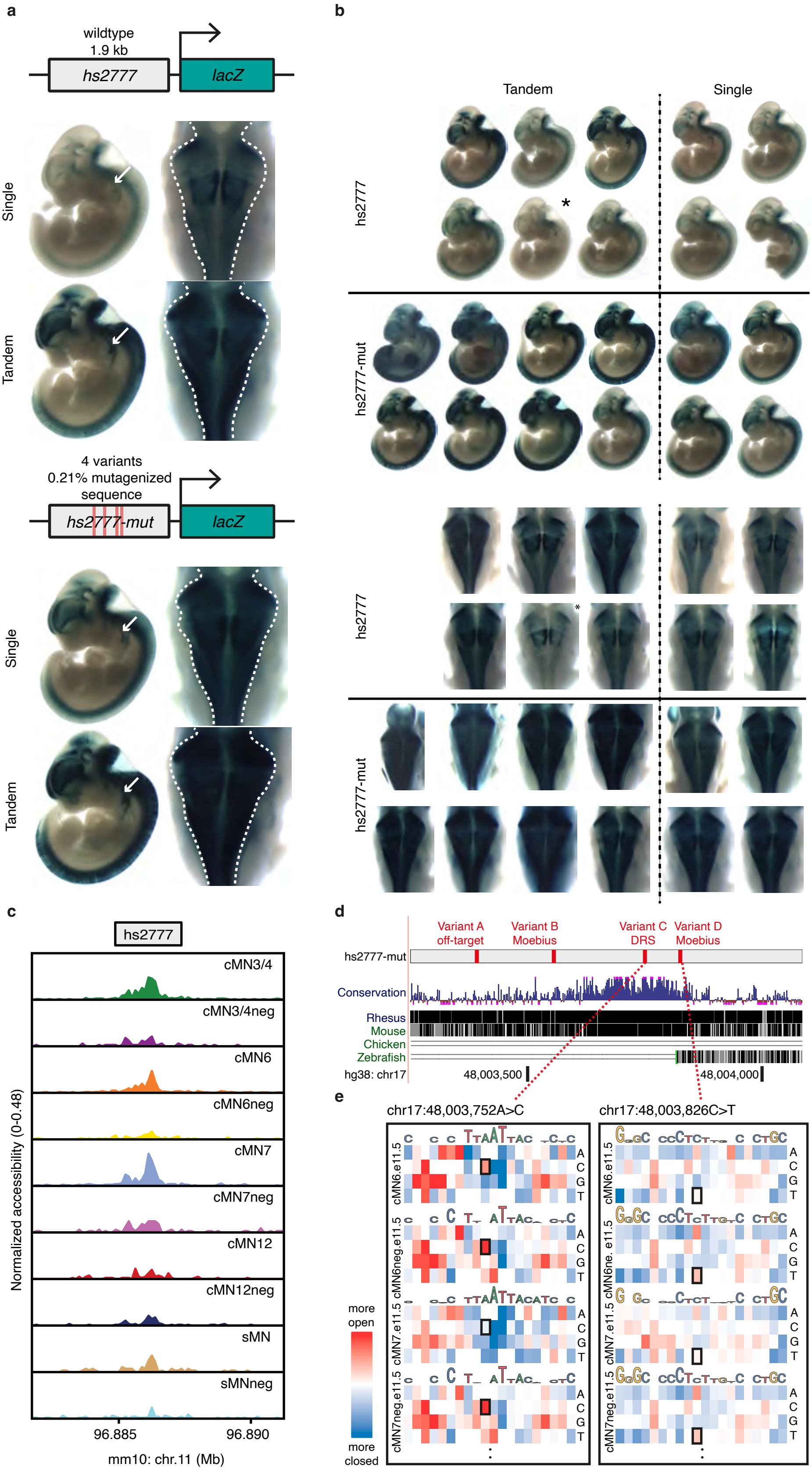

a
wildtype
1.9 kb
hs2777
lacZ
Single
Tandem
4 variants
0.21% mutagenized
sequence
hs2777-mut
lacZ
Single
Tandem
b
Tandem
Single
hs2777
*
hs2777-mut
hs2777
*
hs2777-mut
c
hs2777
cMN3/4
cMN3/4neg
cMN6
cMN6neg
cMN7
cMN7neg
cMN12
cMN12neg
sMN
sMNneg
Normalized accessibility (0-0.48)
96.885
96.890
mm10: chr.11 (Mb)
d
Variant A
off-target
Variant B
Moebius
Variant C
DRS
Variant D
Moebius
hs2777-mut
Conservation
Rhesus
Mouse
Chicken
Zebrafish
hg38: chr17
48,003,500
48,004,000
e
chr17:48,003,752A>C
chr17:48,003,826C>T
cMN6.e11.5
cMN6neg.e11.5
cMN7.e11.5
cMN7neg.e11.5
cMN6ne. e11.5
more
open
more
closed
A
C
G
T

Supplementary Figure 11. Cell type-aware candidate variants alter reporter expression *in vivo.*

a. Representative whole mount *in vivo* enhancer reporter expression for (top) hs2777 wildtype (n = 11) and (bottom) hs2777-mut (n = 15) enhancer constructs. For each reporter insertion, dosage is labelled ("single", "tandem"). Reporter expression views are shown as lateral (left) and dorsal through the 4th ventricle (right). Cranial nerve 7 (white arrows) and surrounding hindbrain tissue (dashed lines) show visible gain of reporter expression.
b. Additional replicates as in (a), matched by injection batch (top and bottom). hs2777-mut constructs reproducibly show increased reporter expression across midbrain, hindbrain, and neural tube. Random insertions are denoted by an asterisk.
c. hs2777 chromatin accessibility profiles in the cranial motor neurons and surrounding cell types. The wildtype element is accessible across multiple cMNs and surrounding cells.
d. UCSC screenshot depicting location of hs2777-mut variants: "Variant A" (chr17:48003393G>A, off-target), "Variant B" (chr17:48003557C>G, Moebius), "Variant C" (chr17:48003752A>C, DRS), and "Variant D" (chr17:48003826C>T, Moebius). hs2777-mut overlaps conserved non-coding sequence, particularly for Variants C and D.
e. Neural net-trained *in silico* saturation mutagenesis predictions for all possible nucleotide changes in hs2777 for selected samples cMN6 e11.5, cMN6neg e11.5, cMN7 e11.5, and cMN7neg e11.5. Predicted loss-of-function nucleotide changes are colored in blue and gain-of-function in red. Specific nucleotide changes corresponding to *in vivo* Variants C and D are boxed. Samples marked with "neg" are GFP-negative cells surrounding the motor neurons of interest. All other samples are GFP-positive motor neurons. Variants C and D are predicted to increase accessibility in relevant samples consistent with their corresponding phenotypes; DRS alters cMN6 but not cMN7 development (Variant C), while MBS alters both (Variant D).

Supplementary Table 1. Sampled cranial motor neurons and their cognate genetic disorders.

Supplementary Table 2. Homogeneity, completeness, and Vmeasure values under different conditions.

Supplementary Table 3. Novel tested enhancer candidates selected from scATAC peaks.

Supplementary Table 4. Permutation test overlap between candidate variants and disease-relevant peaks.

Supplementary Table 1. Sampled cranial motor neurons and their cognate genetic disorders.

Sampled cMN	Mature nerve	Cognate genetic disorder(s)	Phenotype MIM number(s)	Known mode(s) of inheritance	Known coding genes (OMIM)	Linkage regions (OMIM)	Phenotype summary (all are congenital and nonprogressive)
cMN3	Oculomotor	Ptosis, CFEOM1, CFEOM3A, CFEOM3B, CFEOM4, CFEOM5	178300, 300245, 135700, 600638, 609428, 610004, 615065	AD, AR, XLD	*KIF21A, TUBB3, COL25A1, ECEL1, TUBA1A, TUBB2B*	*chr1p34.1-p32, chrXq24-q27.1, chr21q22*	The affected eye has blepharoptosis, restricted vertical eye movements, often restricted horizontal eye movements. Primary globe position is down and often down and out.
cMN4	Trochlear	Fourth nerve palsy	136480	Sporadic	-	-	The affected eye cannot turn inward (adduction) and down.
cMN3/4	Oculomotor + Trochlear	CFEOM2	602078	AR	*PHOX2A*	*chr11q13.4*	The affected eye has blepharoptosis, restricted movements, and is typically fixed in abduction.
cMN6	Abducens	6th nerve palsy, horizontal gaze palsy, Duane retraction syndrome	126800, 604356, 617041, 601536, 607343, 608630, 107480, 603808, 301041	AD, AR	*CHN1, EBF3, MAFB, HOXA1, SALL4, SALL1, MED13, MN1, ZC4H2*	*chr8q13*	6th nerve palsy: the affected eye cannot abduct. HGP: the affected eye cannot abduct or adduct. DRS (type 1): the affected eye cannot abduct (with or without limited adduction) and there is globe retraction on attempted adduction.
cMN7	Facial	Congenital facial weakness	601471, 604185, 614744	AD, AR	*HOXB1, HCFP1 (noncoding)*	*chr3q21-q22, chr10q21.3-q22.1, chr17q21.32*	Unilateral or bilateral facial weakness often resulting in incomplete eye closure, failure to wrinkle forehead, and inability to smile, pucker lips, suck from straw, etc.
cMN6/cMN7	Abducens + Facial	Moebius syndrome	157900	Sporadic	-	*chr13q12.2-q13*	Combination of limited ocular abduction and facial weakness
cMN12	Hypoglossal	Not previously described	-	-	-	-	Weakness and wasting of tongue on one or both sides

Supplementary Table 2. Homogeneity, completeness, and Vmeasure values under different conditions.

cluster type, K[1]	class, c[2]	Subset of data	# cells, N	homogeneity, h	completeness, c	Vmeasure
cluster	sample	all	86089	0.58	0.52	0.55
cluster	time	all	86089	0.31	0.09	0.13
cluster	sample+time	all	86089	0.53	0.58	0.55
cluster	sample	GFPpos	49708	0.84	0.51	0.64
cluster	sample	GFPneg	36381	0.16	0.17	0.17
cluster	sample+time	GFPpos	49708	0.74	0.61	0.67
cluster	sample+time	GFPneg	36381	0.15	0.21	0.17
cluster	sample	GFPpos, e10.5	22337	0.85	0.51	0.64
cluster	sample	GFPneg, e10.5	16463	0.22	0.19	0.2
cluster	sample	GFPpos, e11.5	27371	0.85	0.57	0.68
cluster	sample	GFPneg, e11.5	19918	0.17	0.19	0.18
subcluster	sample+time	all	86089	0.69	0.45	0.54
subcluster	sample+time	GFPpos	49708	0.87	0.46	0.6
subcluster	sample+time	GFPneg	36381	0.43	0.26	0.32
subcluster	sample	GFPpos, e10.5	22337	0.9	0.35	0.5
subcluster	sample	GFPneg, e10.5	16463	0.42	0.18	0.25
subcluster	sample	GFPpos, e11.5	27371	0.9	0.38	0.53
subcluster	sample	GFPneg, e11.5	19918	0.39	0.18	0.24

[1]23 major clusters or 132 subclusters

[2]sample – parsed by biological sample; time – parsed by embryonic age; sample+time – parsed by biological sample and embryonic age

Supplementary Table 3. Novel tested enhancer candidates selected from scATAC peaks.

ID	coordinates (hg38)	Encode H3K27Ac	conservation	Human orthologue	sample size	any expression	MN expression	MN territory*
hs2669	chr1:201010407-201013698	yes	high	yes	9	1	0	-
hs2670	chr1:39885397-39888756	yes	high	yes	6	1	0	-
hs2671	chr1:42952130-42955315	yes	high	yes	2	0	0	-
hs2672	chr1:43833738-43836808	yes	high	yes	7	1	1	sMN, cMN12
hs2673	chr1:48916733-48919461	yes	high	yes	9	1	1	cMN3,cMN4,sMN
hs2674	chr10:71083916-71087354	yes	high	yes	8	1	1	cMN3
hs2675	chr11:128531506-128534286	yes	high	yes	7	1	1	sMN
hs2676	chr11:129635985-129639445	yes	high	yes	5	0	0	-
hs2677	chr11:13096564-13099496	yes	high	yes	4	1	1	sMN
hs2678	chr11:72307322-72310280	yes	high	yes	5	1	1	cMN3, cMN7
hs2679	chr12:113761062-113764654	yes	high	yes	5	1	1	cMN3, cMN6
hs2680	chr12:12238954-12242348	yes	high	yes	4	1	1	sMN, cMN3, cMN12
hs2681	chr13:25179098-25182569	yes	high	yes	6	1	0	-
hs2682	chr15:48004701-48007422	yes	high	yes	9	0	0	-
hs2683	chr16:56100867-56103896	yes	high	yes	6	1	1	cMN3
hs2684	chr18:47994861-47997654	yes	high	yes	8	1	0	-
hs2685	chr2:98555327-98558653	yes	high	yes	4	0	0	-
hs2686	chr22:32538024-32540846	yes	high	yes	7	0	0	-
hs2687	chr3:189795907-189799390	yes	high	yes	9	0	0	-
hs2688	chr3:43957489-43960307	yes	high	yes	5	0	0	-
hs2689	chr4:14866205-14869222	yes	high	yes	8	1	0	-
hs2690	chr4:41856127-41859278	yes	high	yes	4	1	0	-
hs2691	chr7:26438808-26442212	yes	high	yes	4	1	1	cMN6
hs2692	chr8:126500104-126503255	yes	high	yes	7	0	0	-
hs2693	chr8:128581342-128584658	yes	high	yes	3	0	0	-
hs2694	chr8:38935721-38938583	yes	high	yes	5	1	1	cMN3, cMN12

*Positive evidence in at least 1 tested embryo with distinct axonal and/or nucleus expression in the appropriate anatomic territory evaluating for expression in cMN3, cMN4, cMN7, cMN12, and sMN (i.e., celltypes assayed by scATAC only)

Supplementary Table 4. Permutation test overlap between candidate variants and disease-relevant peaks.

Disease group	de novo Z-score	de novo p-value*	dominant z-score	dominant p-value*	permutations
CFEOM	16.931	2.00E-04	30.1398	2.00E-04	5000
CFP	2.9959	1.94E-02	32.9874	2.00E-04	5000
DRS	23.7021	2.00E-04	48.2816	2.00E-04	5000
FNP	4.5624	2.00E-04	10.7372	2.00E-04	5000
MGJW	6.4362	2.00E-04	24.9528	2.00E-04	5000
Moebius	15.3846	2.00E-04	-	2.00E-04	5000

*2-sided

Supplementary Note 1. Global chromatin accessibility dynamics during cranial motor neuron development.

A longstanding observation in developing/differentiating cells is that global chromatin accessibility and gene expression tend to decrease over developmental time[1–3]. However, such inferences assume that true global differences in accessibility outweigh generic differences in sample quality[4]. Taking advantage of the homogenous sample and data quality of our cranial motor neuron chromatin accessibility profiles, we tested for global differences in chromatin accessibility in our dataset from e10.5 to e11.5. In order to estimate global accessibility states for all single cells, we first tabulated the number of accessible peaks[4,5], stratified by embryonic dissection date. Both GFP-positive and -negative cells show significantly greater numbers of accessible peaks at e10.5 than e11.5, consistent with a model of progressive chromatin closure (β_{time} = -0.36; p-value < 1×10^{-15}; negative binomial regression). To better account for experimental variation in sequencing coverage and noise between individual cells, we next calculated Fraction of Reads in Peaks (FRiP) for each individual cell and again stratified by dissection date. After accounting for per-cell read depth and peak number, we observe a significant increase in FRiP over time, again consistent with a model of chromatin closure (β_{time} = 0.049; p-value < 1×10^{-15}; linear regression). Next, we decomposed this combined signal by sample dissection identity. We found a consistent difference in FRiP in the expected direction over developmental time across different cell types and biological replicates (Supplementary Figure 1g; p-value = 4×10^{-5}, binomial test). In addition to mean differences in accessibility among samples, we also observed substantial differences in the distributions of global accessibility--most notably for GFP-positive versus -negative cells at e10.5 (F = 1349, p-value = 1.1×10^{-286}, Brown-Forsythe Test Supplementary Figure 1h). Importantly, while such per-sample metrics as peak number, FRiP, and TSS enrichment are strongly correlated with global accessibility, direct measurements[6] are also needed to explicitly account for potential systematic biases in sample quality.

Supplementary References

1. Argelaguet, R. *et al.* Multi-omics profiling of mouse gastrulation at single-cell resolution. *Nature* **576**, 487–491 (2019).
2. Gulati, G. S. *et al.* Single-cell transcriptional diversity is a hallmark of developmental potential. *Science (80-.).* **367**, 405–411 (2020).
3. Lara-Astiaso, D. *et al.* Immunogenetics. Chromatin state dynamics during blood formation. *Science* **345**, 943–949 (2014).
4. Frank, C. L. *et al.* Regulation of chromatin accessibility and Zic binding at enhancers in the developing cerebellum. *Nat. Neurosci.* **18**, 647–656 (2015).
5. Buenrostro, J. D. *et al.* Integrated Single-Cell Analysis Maps the Continuous Regulatory Landscape of Human Hematopoietic Differentiation. *Cell* **173**, 1535-1548.e16 (2018).
6. Chen, X. *et al.* ATAC-see reveals the accessible genome by transposase-mediated imaging and sequencing. *Nat. Methods* **13**, 1013–1020 (2016).
