# Supplementary material for: A cell type-aware framework for nominating non-coding variants in Mendelian regulatory disorders
Source: Nat Commun. 2024 Sep 27;15:8268. doi: 10.1038/s41467-024-52463-7 (PMC11436875; doi:10.1038/s41467-024-52463-7)
Supplement: Supplementary file 3 — Description of Additional Supplementary Files [file 41467_2024_52463_MOESM3_ESM.pdf]

File Name: Supplementary Data 1

Description: Samples and IDs

File Name: Supplementary Data 2

Description: Per-cluster quality metrics and biological annotations.

File Name: Supplementary Data 3

Description: Target gene predictions for the cranial motor neuron enhancers from the VISTA enhancer database.

File Name: Supplementary Data 4

Description: Activity-by-Contact enhancer and target predictions for the cranial motor neurons.

File Name: Supplementary Data 5

Description: CCDD WGS cohort composition.

File Name: Supplementary Data 6

Description: Dominant multi-hit genes.

File Name: Supplementary Data 7

Description: Phenotypic overlap of EBF3 coding/non-coding variant cases with HADDS.

File Name: Supplementary Data 8

Description: Multi-hit peaks.

File Name: Supplementary Data 9

Description: Neural net predicted significant variants.

File Name: Supplementary Data 10

Description: Screened elements for in vivo variant testing.

File Name: Supplementary Data 11

Description: xTEA de novo transposable element insertions in affected individuals.
