# Supplementary material for: A cell type-aware framework for nominating non-coding variants in Mendelian regulatory disorders
Source: Nat Commun. 2024 Sep 27;15:8268. doi: 10.1038/s41467-024-52463-7 (PMC11436875; doi:10.1038/s41467-024-52463-7)
Supplement: Supplementary file 5 — Reporting Summary [file 41467_2024_52463_MOESM5_ESM.pdf]

nature portfolio

Corresponding author(s): Arthur S. Lee, Elizabeth C. Engle

Last updated by author(s): Aug 6, 2024

Reporting Summary

Nature Portfolio wishes to improve the reproducibility of the work that we publish. This form provides structure for consistency and transparency in reporting. For further information on Nature Portfolio policies, see our Editorial Policies and the Editorial Policy Checklist.

Statistics

For all statistical analyses, confirm that the following items are present in the figure legend, table legend, main text, or Methods section.

n/a	Confirmed	
☐	☒	The exact sample size (*n*) for each experimental group/condition, given as a discrete number and unit of measurement
☐	☒	A statement on whether measurements were taken from distinct samples or whether the same sample was measured repeatedly
☐	☒	The statistical test(s) used AND whether they are one- or two-sided<br>*Only common tests should be described solely by name; describe more complex techniques in the Methods section.*
☒	☐	A description of all covariates tested
☐	☒	A description of any assumptions or corrections, such as tests of normality and adjustment for multiple comparisons
☐	☒	A full description of the statistical parameters including central tendency (e.g. means) or other basic estimates (e.g. regression coefficient) AND variation (e.g. standard deviation) or associated estimates of uncertainty (e.g. confidence intervals)
☐	☒	For null hypothesis testing, the test statistic (e.g. *F*, *t*, *r*) with confidence intervals, effect sizes, degrees of freedom and *P* value noted<br>*Give P values as exact values whenever suitable.*
☒	☐	For Bayesian analysis, information on the choice of priors and Markov chain Monte Carlo settings
☒	☐	For hierarchical and complex designs, identification of the appropriate level for tests and full reporting of outcomes
☐	☒	Estimates of effect sizes (e.g. Cohen's *d*, Pearson's *r*), indicating how they were calculated

Our web collection on statistics for biologists contains articles on many of the points above.

Software and code

Policy information about availability of computer code

Data collection	No software or code used to collect data
Data analysis	Code used for analysis is available without restriction at https://github.com/arthurlee617/noncoding-mendel. Published software used for analysis are described in Methods and include ArchR 1.0.0, macs2, sabre 0.4.3, bedtools 2.29.1, star 2.4.0, rsem 1.2.22, seurat v4, signac 1.0.0, DEseq 1.34.0, GATK 4.0, GATK SV, xTea 0.1.2, GenomicRanges 1.54.1, regionR 1.34.0, basenji 0.4, dplyr 1.1.4, tidyr 1.3.1, stringr 1.5.1

For manuscripts utilizing custom algorithms or software that are central to the research but not yet described in published literature, software must be made available to editors and reviewers. We strongly encourage code deposition in a community repository (e.g. GitHub). See the Nature Portfolio guidelines for submitting code & software for further information.

Data

Policy information about availability of data

All manuscripts must include a data availability statement. This statement should provide the following information, where applicable:

- Accession codes, unique identifiers, or web links for publicly available datasets
- A description of any restrictions on data availability
- For clinical datasets or third party data, please ensure that the statement adheres to our policy

All ATAC-seq, RNA-seq, CUT&Tag, and multiome data generated in this work are available without restriction through the Gene Expression Omnibus accession number GSE254090 – bulk ATAC-seq (GSE254083); scATAC-seq (GSE254086); scCUT&Tag (GSE254088); bulk RNA-seq (GSE254084); scRNA-seq (GSE254089); scMultiome (GSE254085). Human WGS data are available through dbGaP authorized access (accession phs001247). In vivo enhancer results are available without

restriction via the VISTA Enhancer Database (https://enhancer.lbl.gov) under identical enhancer IDs referenced in the manuscript.

Research involving human participants, their data, or biological material

Policy information about studies with human participants or human data. See also policy information about sex, gender (identity/presentation), and sexual orientation and race, ethnicity and racism.

Reporting on sex and gender	See dbGaP Study Accession: phs001247. Sex for human participants were collected from clinical information. Reported or genetically inferred sex was used for quality control, relatedness checks, and estimates of contamination. Sex specific traits were not investigated.
Reporting on race, ethnicity, or other socially relevant groupings	See dbGaP Study Accession: phs001247. Reported race or ethnicity was recorded ("White, Hispanic, Black, Asian, multiple race, unknown") and used for genetic ancestry group allele frequency comparisons from genomic population databases.
Population characteristics	See dbGaP Study Accession: phs001247. Study participants were selected for CCDD diagnosis (CFEOM, CP, DRS/HGP, CFP, or Moebius syndrome). Syndromic features affecting other organ systems such as cardiovascular, limb, and skeletal were recorded.
Recruitment	See dbGaP Study Accession: phs001247. Study participants were recruited based on CCDD diagnosis. Self selection would occur for patients and/or their families unwilling to participate in genetic research. Such self selection would reduce sample sizes.
Ethics oversight	Boston Children's Hospital Institutional Review Board clinicaltrials.gov identifier NCT03059420

Note that full information on the approval of the study protocol must also be provided in the manuscript.

Field-specific reporting

Please select the one below that is the best fit for your research. If you are not sure, read the appropriate sections before making your selection.

- [x] Life sciences
- [ ] Behavioural & social sciences
- [ ] Ecological, evolutionary & environmental sciences

For a reference copy of the document with all sections, see nature.com/documents/nr-reporting-summary-flat.pdf

Life sciences study design

All studies must disclose on these points even when the disclosure is negative.

Sample size	Sample sizes were determined by availability of CCDD study participants. No other statistical methods were used to predetermine sample size.
Data exclusions	Samples were excluded if they didn't meet multiple established standards for data quality, including DNA fragmentation distribution, DNA concentration, unique barcodes, PCR duplicates, library complexity, and others.
Replication	Multiple biological replicates were collected for each sample and multiple measures of sample concordance were verified.
Randomization	All usable embryos of a given genotype were collected.
Blinding	Comparisons across different genotypes were not blinded but identical pre-determined benchmarks of data quality were applied and final readout tabulation did not require subjective operator judgment.

Reporting for specific materials, systems and methods

We require information from authors about some types of materials, experimental systems and methods used in many studies. Here, indicate whether each material, system or method listed is relevant to your study. If you are not sure if a list item applies to your research, read the appropriate section before selecting a response.

Materials & experimental systems

n/a	Involved in the study	
[ ]	[x]	Antibodies
[x]	[ ]	Eukaryotic cell lines
[x]	[ ]	Palaeontology and archaeology
[ ]	[x]	Animals and other organisms
[ ]	[x]	Clinical data
[x]	[ ]	Dual use research of concern
[x]	[ ]	Plants

Methods

n/a	Involved in the study	
[x]	[ ]	ChIP-seq
[ ]	[x]	Flow cytometry
[x]	[ ]	MRI-based neuroimaging

Antibodies

Antibodies used	1:50 H3K27Ac primary antibody (monoclonal Rabbit anti-mouse, Abcam ab177178), 1:50 IgG secondary antibody (guinea pig anti-rabbit Novus Biologicals, NBP1-72763), 1:20 pAG-Tn5 (EpiCypher 15-1017)
Validation	Our protocol is based on a published protocol using identical validated antibodies (https://doi.org/10.1038/s41587-021-00869-9)

Animals and other research organisms

Policy information about studies involving animals; ARRIVE guidelines recommended for reporting animal research, and Sex and Gender in Research

Laboratory animals	C57BL/6 JAX #000664, 129S1/C57BL/6J IslMN:GFP JAX #017952, Hb9:GFP JAX #005029, FVB/NJ JAX #001800, cRE2Fam4/Fam4, cRE2Fam5/Fam5. Mice were maintained in pathogen-free environments and fed ad libitum with sterile standard diet and water in a temperature, humidity, and light-controlled rooms (22°C set-point +/- 1.3°C, RH35-70% +/- 5%, 12/12 light/dark cycle, 10-15 air changes per hour). Fam4 and Fam5 snv/snv mice were maintained on a 129S1/C57BL/6J mixed background. Experimental snv/snv mice were generated by intercrossing snv/snv breeders.
Wild animals	none
Reporting on sex	Sex was not considered in study design
Field-collected samples	none
Ethics oversight	Institutional Animal Care and Use Committees of Boston Children's Hospital (Protocol number 00001852)

Note that full information on the approval of the study protocol must also be provided in the manuscript.

Clinical data

Policy information about clinical studies

All manuscripts should comply with the ICMJEguidelines for publication of clinical research and a completedCONSORT checklist must be included with all submissions.

Clinical trial registration	clinicaltrials.gov identifier NCT03059420
Study protocol	see https://clinicaltrials.gov/ct2/show/NCT03059420
Data collection	Participants were enrolled between ~1990-present and data were collected in both clinical and research settings.
Outcomes	Primary outcomes are provied at https://clinicaltrials.gov/ct2/show/NCT03059420.

Plants

Seed stocks	none
Novel plant genotypes	none
Authentication	none

Flow Cytometry

Plots

Confirm that:

- [x] The axis labels state the marker and fluorochrome used (e.g. CD4-FITC).
- [x] The axis scales are clearly visible. Include numbers along axes only for bottom left plot of group (a 'group' is an analysis of identical markers).
- [x] All plots are contour plots with outliers or pseudocolor plots.
- [x] A numerical value for number of cells or percentage (with statistics) is provided.

Methodology

Sample preparation	Pregnant females were sacrificed at 10.5 or 11.5 days post-conception and whole embryos were grossly dissected in chilled 1x PBS (ThermoFisher) then immediately placed in 1x B27 supplement (Gibco 17504044) in Hibernate E (Fisher NC0285514). Next, GFP-positive cranial motor neurons, GFP-positive spinal motor neurons, and GFP-negative surrounding cells were microdissected in pre-chilled HBSS (ThermoFisher) and placed in 1x B-27 supplement, 1x Glutamax (ThermoFisher 35050061), and 100 U/mL Penicillin-Streptomycin (PenStrep, ThermoFisher 15140122) in Hibernate E (medium 2). Microdissected tissues were dissociated using papain and ovomucoid solutions prepared from Papain Dissociation System (Worthington Biochemical LK003150). Tissues were resuspended in papain solution. Samples were then incubated at 37°C for 30 minutes and agitated every 10 minutes to ensure complete dissociation. Following incubation, samples were spun down at 300 rcf for 5 minutes, the supernatant was removed, and dissociated tissues were resuspended in 500 uL of ovomucoid solution (plus or minus 100 µL depending on quantity of tissue). Tissues were again spun down at 300 rcf for 5 minutes and resuspended in 500 µL of medium 2 (plus or minus 100 µL depending on quantity of tissue) and transferred to a 5mL polystyrene round bottom tube on ice.
Instrument	ARIA-561 FACS machine at the Immunology Research Core at Harvard Medical School and BD FACS Aria II at the Jimmy Fund Core at the Dana-Farber Cancer Institute
Software	FlowJo Analysis Software
Cell population abundance	After gating on size, granularity, doublets, and fluorescence, approximately 1% of the initial dissociated sample is collected . Purity is established based on morphology, fluorescence, and chromatin/gene expression profiles.
Gating strategy	Forward scatter area (FSC-A) and side scatter area (SSC-A), corresponding to cell size and granularity/complexity, are used to enrich for intact cells and exclude debris. Forward scatter width (FSC-W) and FSC-A are used to exclude doublets. GFP-positive and -negative gating thresholds are determined by comparing samples to dissociated limb buds as negative controls.

☒ Tick this box to confirm that a figure exemplifying the gating strategy is provided in the Supplementary Information.
